# Axonal Synapses Utilize Multiple Synaptic Ribbons in the Mammalian Retina

**DOI:** 10.1371/journal.pone.0052295

**Published:** 2012-12-17

**Authors:** Hong-Lim Kim, Ji Hyun Jeon, Tae-Hyung Koo, U-Young Lee, Eojin Jeong, Myung-Hoon Chun, Jung-Il Moon, Stephen C. Massey, In-Beom Kim

**Affiliations:** 1 Department of Anatomy, College of Medicine, The Catholic University of Korea, Seoul, Korea; 2 Integrative Research Support Center, College of Medicine, The Catholic University of Korea, Seoul, Korea; 3 Department of Ophthalmology, College of Medicine, The Catholic University of Korea, Seoul, Korea; 4 Catholic Institute for Applied Anatomy, College of Medicine, The Catholic University of Korea, Seoul, Korea; 5 Department of Ophthalmology and Visual Sciences, University of Texas Health Science Center, Houston, Texas, United States of America; Virginia Tech Carilion Research Institute, United States of America

## Abstract

In the mammalian retina, bipolar cells and ganglion cells which stratify in sublamina *a* of the inner plexiform layer (IPL) show OFF responses to light stimuli while those that stratify in sublamina *b* show ON responses. This functional relationship between anatomy and physiology is a key principle of retinal organization. However, there are at least three types of retinal neurons, including intrinsically photosensitive retinal ganglion cells (ipRGCs) and dopaminergic amacrine cells, which violate this principle. These cell types have light-driven ON responses, but their dendrites mainly stratify in sublamina *a* of the IPL, the OFF sublayer. Recent anatomical studies suggested that certain ON cone bipolar cells make axonal or ectopic synapses as they descend through sublamina *a*, thus providing ON input to cells which stratify in the OFF sublayer. Using immunoelectron microscopy with 3-dimensional reconstruction, we have identified axonal synapses of ON cone bipolar cells in the rabbit retina. Ten calbindin ON cone bipolar axons made *en passant* ribbon synapses onto amacrine or ganglion dendrites in sublamina *a* of the IPL. Compared to the ribbon synapses made by bipolar terminals, these axonal ribbon synapses were characterized by a broad postsynaptic element that appeared as a monad and by the presence of multiple short synaptic ribbons. These findings confirm that certain ON cone bipolar cells can provide ON input to amacrine and ganglion cells whose dendrites stratify in the OFF sublayer *via* axonal synapses. The monadic synapse with multiple ribbons may be a diagnostic feature of the ON cone bipolar axonal synapse in sublamina *a*. The presence of multiple ribbons and a broad postsynaptic density suggest these structures may be very efficient synapses. We also identified axonal inputs to ipRGCs with the architecture described above.

## Introduction

Parallel processing of sensory information is defined as the simultaneous processing of sensory information through many independent circuits and it is considered to be a common strategy in the mammalian brain. In the retina, many aspects of an image, such as contour, contrast, color and movement, are processed in parallel. Bipolar cells, the second order neurons of the retina, play a central role in this parallel processing because the photoreceptor signals immediately diverge into multiple bipolar cell pathways [1]–[4]. There are ≈10 types of cone bipolar cells and one type of rod bipolar cell in the mammalian retina [5]–[9]. Based on their light response polarity, they are functionally divided into OFF and ON bipolar cells [1], [10]. Since the time of Cajal, the inner plexiform layer (IPL) has traditionally been divided into 5 different strata numbered 1 through 5. OFF bipolar axons ramify in strata 1 and 2, known as sublamina *a* or the OFF sublayer, where they synapse onto dendrites of amacrine cells and ganglion cells showing OFF responses. In contrast, ON bipolar axons make synaptic contacts with ON driven third order neurons in strata 3–5, known as sublamina *b* or the ON sublayer, in the inner half of the IPL [6], [10]–[14]. Ganglion cells with ON and OFF responses, such as ON/OFF directionally selective ganglion cells, are bistratified with dendrites in both sublamina *a* and sublamina *b* [15]. This stunning relationship between anatomy and physiology is a key principle of retinal circuit organization.

However, there are at least three types of inner retinal neurons that violate this principle. Melanopsin-expressing intrinsically photoresponsive ganglion cells (ipRGCs) [16]–[19], dopaminergic amacrine cells (DACs) [20], [21], and bistratified diving ganglion cells [22] show ON responses to light despite their main stratification in the OFF sublayer. Thus, they apparently break the stratification rules of the IPL. Previous electron microscopy (EM) work has shown that the dopaminergic amacrine cells receive bipolar inputs in stratum 1 [23], [24]. In more recent anatomical studies, the presence of both pre- and post-synaptic markers at the contact sites between descending ON bipolar axons and the three cell types with paradoxical light responses suggested that certain ON cone bipolar cells can provide ON inputs to all three cell types as they traverse sublamina *a* [25]–[27]. Nevertheless, EM studies are needed to examine the architecture of these synapses.

In this study, we identified axonal synapses of ON cone bipolar cells in sublamina *a*, the OFF sublayer of the IPL, and described the characteristics which distinguished them from the ribbon synapses formed at bipolar axon terminals in sublamina *b* of the IPL. Specifically, we labeled the axons of the calbindin ON cone bipolar cells in the rabbit retina and reconstructed their axonal synapses in sublamina *a* of the IPL by EM with 3-dimensional (3D) reconstruction using serial ultrathin sections. All the calbindin ON cone bipolar cells examined in this study made axonal ribbon synapses onto amacrine cell and ganglion cell dendrites in sublamina *a* of the IPL, in an *en passant* manner. Unlike the dyads at ribbon synapses formed by axon terminals in sublamina *b*, these axonal ribbon synapses were exclusively monadic. The axonal synapses were characterized by multiple short synaptic ribbons and broad postsynaptic densities. The monadic ribbon synapse may be a diagnostic feature of ON cone bipolar axonal synapses in sublamina *a*.

## Results

### Immunofluorescence: Calbindin ON Cone Bipolar Cells Make Axonal Synapses in Sublamina *a* of the IPL

**Figure 1:**
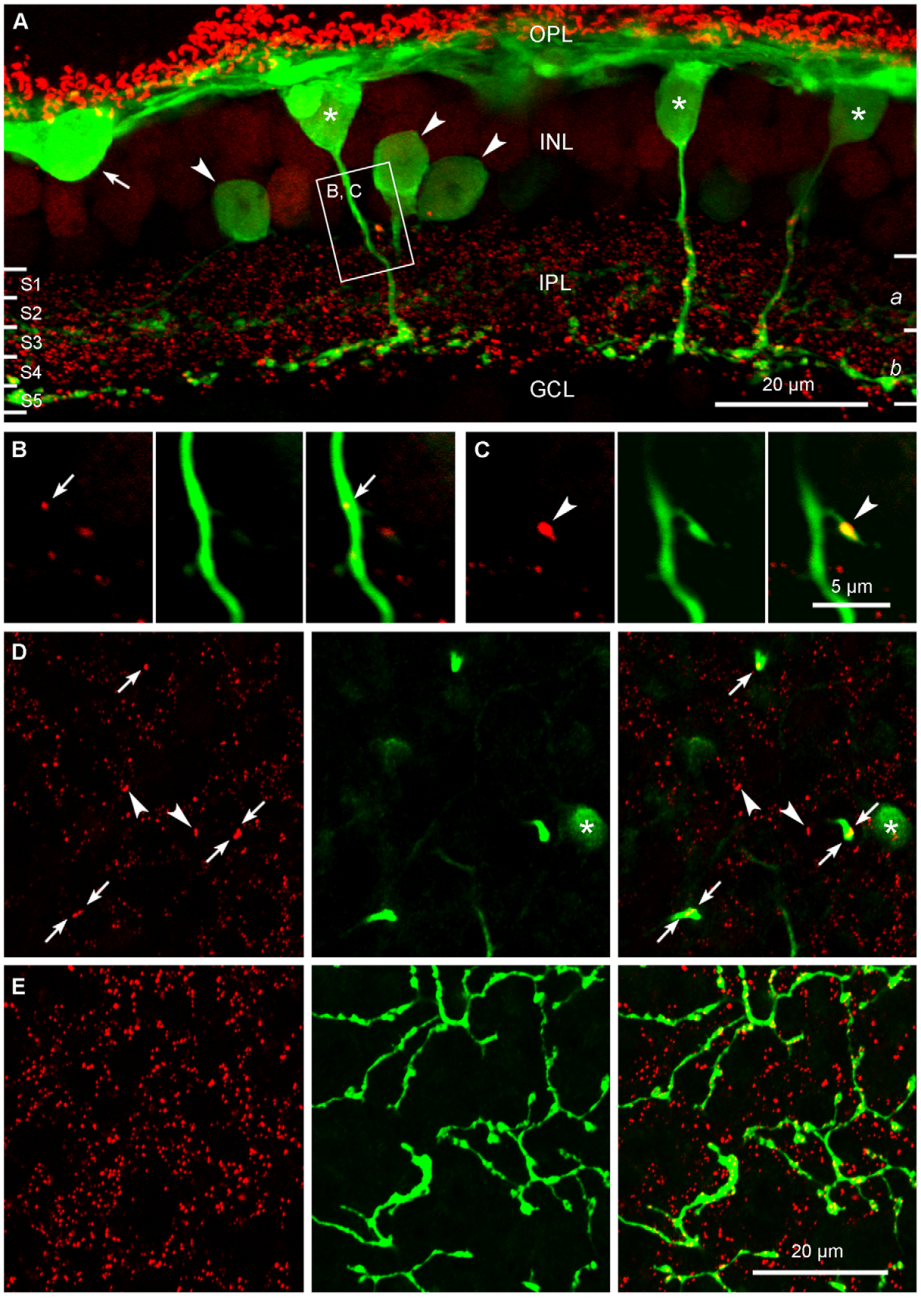
Confocal microscopic evidence that calbindin ON cone bipolar cells make axonal synapses in the IPL. A: An antibody to calbindin (green) labels a horizontal cell (arrow), three amacrine cells (arrowheads), and 3 bipolar cells (asterisks) in the INL. The axons of these calbindin labeled bipolar cells ramify in the border between strata 4 and 5 of sublamina *b* of the IPL, indicating that the calbindin labeled cells are ON-type cone bipolar cells. RIBEYE (red) labeled synaptic ribbons are seen in the OPL as horseshoes and in the IPL as puncta. The boxed area is magnified in B and C. B, C: Magnified images are taken from two 0.38-μm-thick single optical sections. A calbindin ON cone bipolar descending axon contains two synaptic ribbons in stratum 1. One is localized at the main axonal stem (arrow in B) and the other is localized on a small branch from the main descending axon (arrowhead in C). D, E: Both confocal micrographs were taken at the same location from a wholemount piece of the retina. The focal planes for D and E are in sublaminae *a* and *b* of the IPL, respectively. Three calbindin ON cone bipolar descending axons and an amacrine soma (asterisk) are seen in D. All of the labeled axons contain at least one RIBEYE labeled synaptic ribbon (arrows). Arrowheads indicate putative axonal synaptic sites formed by additional ON bipolar cell types. In E, numerous synaptic ribbons are found in calbindin ON cone bipolar axon terminals. In addition, there are many synaptic ribbons from other bipolar cells.

The calbindin antibody labels a variety of neurons in the rabbit retina including horizontal cells, a subset of ON cone bipolar cells, referred to as calbindin bipolar cells, and a few wide-field amacrine cells [28]. Figure 1A shows a confocal stack, 4 µm thick, from a 40 µm thick vibratome section of the rabbit retina double-labeled for calbindin (green) and RIBEYE (red), a synaptic ribbon marker. A band of horizontal cell processes in the outer plexiform layer (OPL) and a horizontal cell body (arrow) were heavily stained. Three amacrine cells (arrowheads), adjacent to the inner nuclear layer (INL) contributed a band of processes to stratum 3 in the middle of the inner plexiform layer (IPL). In addition, three calbindin bipolar cells (asterisks) had axons which passed through sublamina *a* to terminate in a narrow band of axon terminals at the border of strata 4 and 5 in sublamina *b* (Fig. 1A), as previously reported [26], [28].

As expected, synaptic ribbons were distributed throughout the OPL and IPL. The photoreceptor ribbons in the OPL were particularly bright and prominent. The large horseshoe-shaped ribbons are contained within rod spherules. Synaptic ribbons in the inner retina were much smaller, appearing as small puncta distributed evenly throughout the IPL. Many synaptic ribbons were associated with the axon terminals of calbindin bipolar cells in sublamina *b*. Interestingly, some of the largest RIBEYE labeled structures were colocalized with the axons of calbindin bipolar cells as they traversed sublamina *a*. Most of the smaller ribbons in sublamina *a* were not colocalized with calbindin bipolar axons and may be associated with OFF bipolar cells. Consistent with a previous report [26], we found two distinct structures associated with synaptic ribbons and ON bipolar axons in sublamina *a*. In the first type, the ribbons were located within the trunk of the descending axon (Fig. 1B). These we refer to as axonal ribbons or *en passant* synapses. Secondly, sometimes a very fine branch left the axon in sublamina *a* ending in a small swelling containing a synaptic ribbon (Fig. 1C). Both structures have been called ectopic synapses and they contribute synaptic ribbons at the same level in sublamina *a*, predominantly in stratum 1 of rabbit or mouse retina [25], [26].

In wholemount retina (Fig. 1D), with the focus in sublamina *a*, three calbindin bipolar axons appeared as small dots if they were normal to the section plane or short processes if they were oblique. The soma of a calbindin labeled amacrine cell (asterisk) was also present indicating the level of focus was close to the border with the INL. All three calbindin bipolar axons (arrows) contained one or more axonal ribbons. This is consistent with previous work that showed nearly 90% of the calbindin bipolar axons contained synaptic ribbons [26]. In addition, there were additional large synaptic ribbons (arrowheads) that were not colocalized with calbindin bipolar axons. If these large RIBEYE stained structures are axonal ribbons, as opposed to the smaller synaptic ribbons not associated with bipolar axons, this suggests that one or more additional ON bipolar cell types may make axonal ribbons as they cross sublamina *a*. Following the calbindin labeled axons down to strata 4/5 (Fig. 1E), showed the calbindin bipolar axon terminals, which had a uniform coverage at this depth. The calbindin bipolar terminals contained many synaptic ribbons but there were many additional RIBEYE labeled profiles in the IPL. These represent synaptic ribbons from other ON bipolar cell types or rod bipolar cells that are also found at this level.

### Axonal Ribbon Synapses of ON Cone Bipolar Cell Have a Single Postsynaptic Target

**Figure 2:**
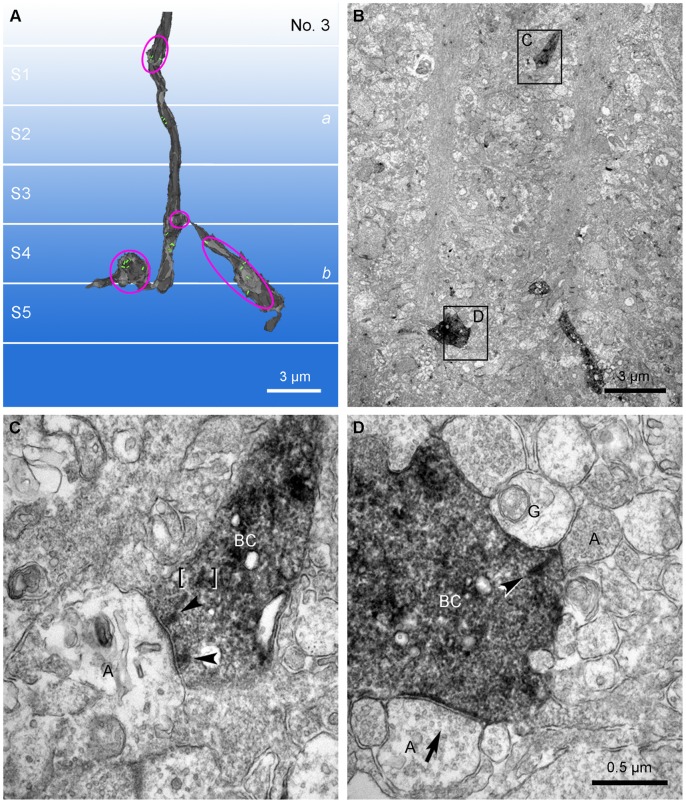
A comparison of axonal synapses and axon terminal synapses of the calbindin ON cone bipolar cell. A: The 3D reconstruction model for No. 3 calbindin ON cone bipolar cell axon (Fig. 3; Table 1). Magenta ellipses are the labeled profiles shown in B. B: Low magnification view of an electron micrograph used to make the 3D reconstruction model shown in A. Four immunolabeled axonal parts are seen. Among them, one (C) in the upper part of the figure is located in stratum 1 and the remaining three in the lower part are located in strata 4 and 5. The descending axonal part in stratum 1 and an axon terminal part in stratum 5 are depicted and magnified in C and D, respectively. C: The labeled axon descends obliquely to form an axonal ribbon synapse onto an amacrine process (A) represented as a monad. At this synapse, two synaptic ribbons (arrowheads) are engaged. Brackets indicate a putative ribbon in transport. D: A calbindin labeled bipolar axon terminal with a synaptic input from an amacrine cell (A) (arrow) forms a ribbon synapse (arrowhead) onto a postsynaptic dyad composed of a ganglion dendrite (G) and an amacrine process (A).

In vertical ultrathin sections of the rabbit retina, the axons of calbindin bipolar cells could be easily identified by their peroxidase reaction products (Fig. 2, S1). Amacrine and ganglion cell dendrites were identified according to ultrastructural criteria [29]–[32]. That is, the amacrine processes were filled with synaptic vesicles and made conventional chemical output synapses, while ganglion cell dendrites contained microtubules and microfilaments instead of synaptic vesicles. As expected from the confocal observations, ribbon synapses were found on the descending axons of calbindin ON cone bipolar cells in sublamina *a* of the IPL (Fig. 2C). Thus, we have confirmed the presence of axonal synapses made by ON cone bipolar cells in the OFF sublayer.

**Figure 3:**
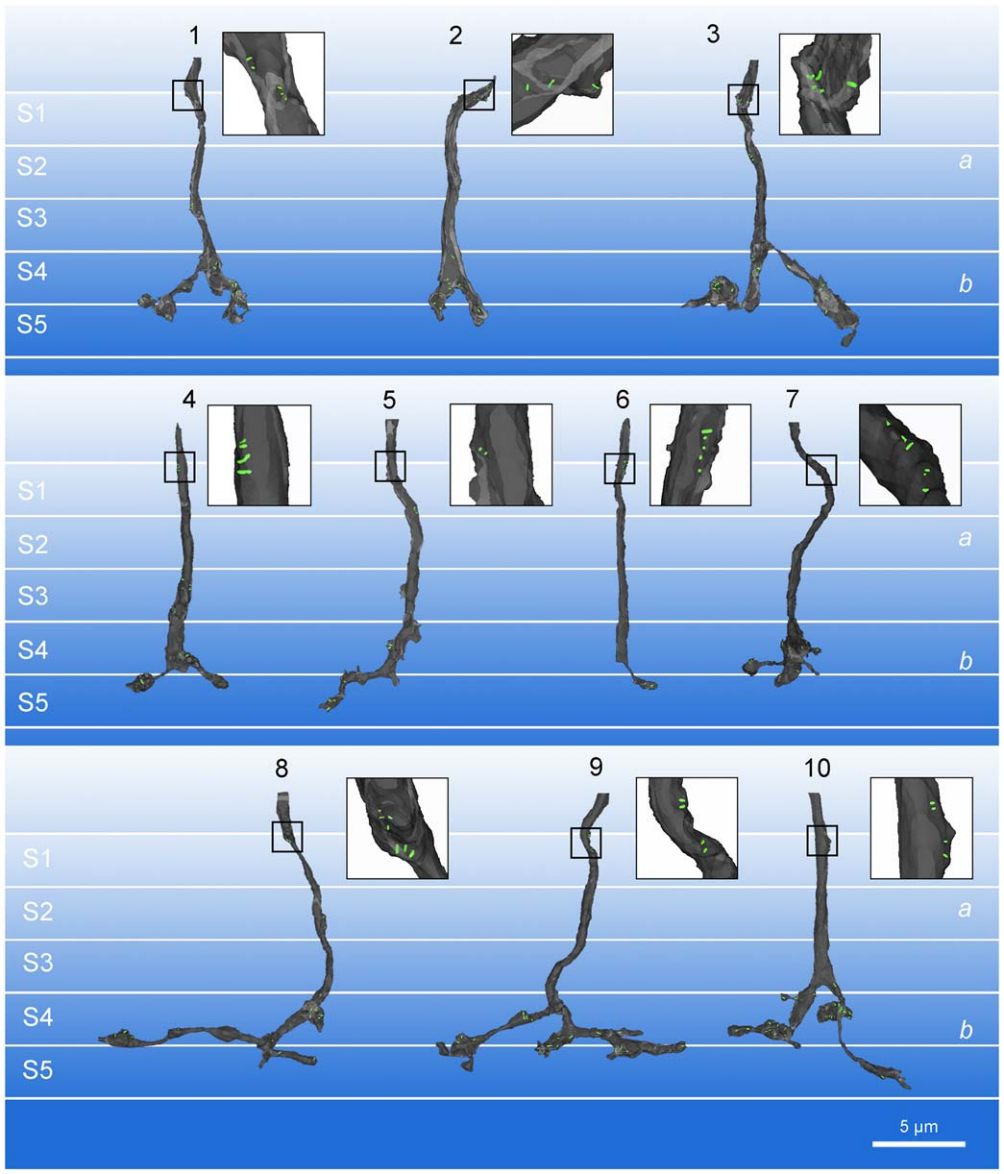
The 3D reconstruction of calbindin ON cone bipolar cell axons in the IPL. Ten reconstructed models of calbindin bipolar axons are demonstrated. Insets: Higher magnification views of calbindin ON cone bipolar axons at the border of the INL and the IPL. Insets show that all calbindin bipolar cells contain multiple synaptic ribbons in this IPL region.

Ten calbindin ON cone bipolar axons within the IPL were partially reconstructed using serial ultrathin sections (Fig. 3). All of descending axons examined formed axonal synapses mostly in the outermost IPL close to the INL. In stratum 1 of sublamina *a*, the synaptic ribbons were localized close and arranged perpendicular to the presynaptic axonal membrane (Fig. 2C, S1), suggesting a functional ribbon involved in synaptic transmission. We found 19 ribbon synapses onto the postsynaptic targets (amacrine and ganglion cells) in stratum 1 of the IPL (Table 1) and thus, a calbindin ON cone bipolar cells made on average 1.9 axonal synapses in this region. In a few cases, 3/3 calbindin ON cone bipolar cells, we also found axonal ribbons lower in stratum 2. While this still belongs to sublamina *a*, we excluded these few ribbons from our analysis because their depth may indicate a different post-synaptic target.

**Table 1:** Synapses made by calbindin ON cone bipolar axons in sublamina *a* of the IPL.^a^

Bipolar cell no.	1	2	3	4	5	6	7	8	9	10	Total
Total ribbons	5	3	6	4	2	5	6	7	4	4	46
*Synaptic output*											
AC monad	1	1	2			1	1	3	2		11
GC monad	1	1		1	1	1	1			2	8

a Abbreviations: AC: amacrine cell; GC: ganglion cell.

A few ribbons, with typical electron dense staining, uniform thickness and homogenous contrast were found in the middle of the axon, arranged parallel to and distant from the axonal membrane (Fig. 2C, S1). This suggests a ribbon in transport from the soma to the axon terminal or other synaptic sites. To exclude ribbons in transport, we made the criteria for “functional” or “synaptic” ribbon in sublamina *a* as follows: the ribbon is arranged perpendicular to the axonal membrane and localized to be opposed to the postsynaptic density area of the synaptic target. In this study, only functional ribbons were counted and represented in 3D models (Fig. 3; Table 1).

To determine the unique features of the axonal ribbon synapses made by ON bipolar cells in sublamina *a*, we compared the axonal ribbon synapses to the ribbon synapses made by ON bipolar cell axon terminals in sublamina *b* (Fig. 2). As a general rule, the axon terminal synaptic ribbon has two postsynaptic elements (known as a dyad) (Fig. 2D). We have previously reported that calbindin bipolar axon terminals make ribbon synapses in sublamina *b* at postsynaptic dyads, which are mainly composed of an amacrine cell process and a ganglion cell dendrite or two amacrine processes [33]. In contrast, at each axonal ribbon synapse (n = 19) from 10 calbindin ON cone bipolar cells, there was only one postsynaptic element. Synapses with this structure are known as monads (Fig. 2C, S1; Table 1). The postsynaptic component of the monad was either a ganglion cell dendrite or an amacrine process. Numerically, amacrine processes (n = 11/19) were encountered slightly more than ganglion cell dendrites (n = 8/19) as postsynaptic elements.

### Additional Features of the Axonal Synapses: Multiple Short Ribbons and Broad Area of Postsynaptic Density

Another reliable characteristic of axonal synapses was easily identified by electron microscopy. In this series of reconstructed calbindin bipolar cells, we found two or more synaptic ribbons at most of the monads formed by axonal synapses (16 of 19; 89%) (Fig. 2C, S1; Table 1). A total of 46 synaptic ribbons were observed in 19 synaptic monads and thus each synapse contained an average of approximately 2.4 ribbons, with a maximum 5.

**Figure 4:**
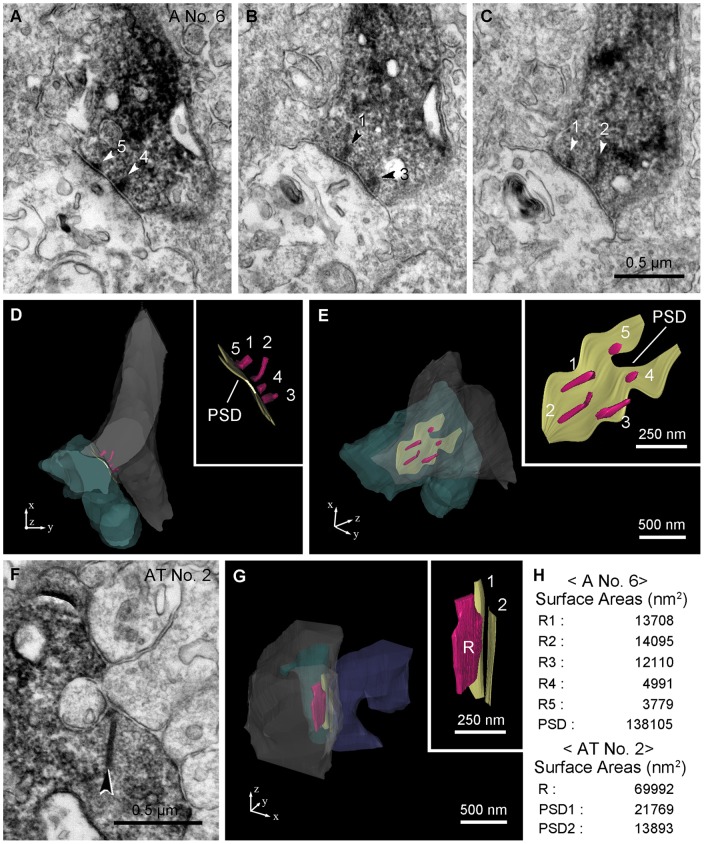
3D reconstruction of axonal and axon terminal synapses of a calbindin ON cone bipolar cell. A–E: The 3D reconstruction of No. 6 axonal (A) synapse was derived from electron micrographs. A–C: Three electron micrographs of a calbindin ON cone bipolar axonal synapse show five synaptic ribbons (1–5, arrowheads) and a broad postsynaptic density on a postsynaptic amacrine process. D, E: Reconstructed synaptic ribbons (R, maroon) of a calbindin bipolar axon (gray) and the postsynaptic density (PSD, khaki) of an amacrine process (sea green) are displayed at two different angles and insets show magnified synaptic ribbons and the postsynaptic density. F, G: 3D reconstruction of No. 2 axon terminal (AT) synapse was derived from electron micrographs. F: An electron micrograph shows a calbindin ON cone bipolar axon terminal synapse containing a synaptic ribbon (arrowhead) and its postsynaptic dyad. G: A synaptic ribbon (R) of a calbinidn bipolar axon terminal (gray) and two postsynaptic densities (1, 2) of the postsynaptic elements composed of an amacrine (state blue) and a ganglion (sea green) are seen. H: Data from image analysis of surface area of the synaptic ribbon and the postsynaptic density from No. 6 axonal synapse (D, E) and No. 2 axon terminal synapse. The table shows that individual axonal ribbons are smaller but the post-synaptic density at axonal ribbons is much larger compared to the axon terminal synapses.

In addition, axonal synapses had relatively short ribbons and a broad postsynaptic density area (Fig. 2). We measured the maximum length of each synaptic ribbon from vertical ultrathin sections and estimated the ribbon plate surface area, and the postsynaptic density area from reconstructed 3D models of both axonal (n = 7) and axon terminal (n = 7) ribbon synapses (Fig. 4; Table 2). Compared to those of the axon terminal ribbon synapse, the average length of individual synaptic ribbon and the ribbon plate surface area of the axonal synapse was 53.7% (108 nm/201 nm) and 20.4% (9353 nm^2^/45795 nm^2^), respectively. In this group of 7 axonal synapses (Table 2), there were 24 ribbons, an average of 3.4 ribbons/axonal synapse. Multiplying the average ribbon area by the number of ribbons gave a total ribbon plate surface area of 32066 nm^2^, approximately 70% of the value for axon terminal ribbons.

**Table 2:** Comparison of axonal and axon terminal ribbon synapses of the calbindin ON cone bipolar cell in length and surface area of the synaptic ribbon and in postsynaptic density area.

		Axonal Ribbon Synapse (n = 7)	Axon Terminal Ribbon Synapse (n = 7)
Presynaptic	Total Ribbons	24	7
	Average of Individual Maximum Ribbon Length on the Vertical Section (nm)	108	201
	Average of Individual Ribbon Surface Area (nm^2^)	9353	45795
	Average of Ribbon Surface Area per Synapse (nm^2^)	32066	45795
Postsynaptic	Total Postsynaptic Elements	7	14
	Average of Postsynaptic Density Area (nm^2^)	101637	17451

The post-synaptic density was easily identified by membrane that was thicker and more electron-dense than other sections of cell membrane. In addition, it had fluffy extensions toward postsynaptic elements and it was apposed to the synaptic ribbon(s). At axonal synapses, the area of the postsynaptic density (101637 nm^2^) was much greater than that of the conventional ribbon synapse (17451 nm^2^; ratio 5.8) (Table 2). This mismatch between presynaptic element and postsynaptic element could be compensated by multiple ribbon engagement. Thus, when the axonal postsynaptic density was normalized by the number of synaptic ribbons (3.4), and the axon terminal postsynaptic area was multiplied by 2 for the dyad structure, the axonal ribbons had 29,893 nm^2^/ribbon compared to 34,902 nm^2^/ribbon, ratio 0.86, close to the ratio for total ribbon area, 0.7. Thus, area of the post-synaptic density may be related to the ribbon number.

### Reconstructions of Additional ON Cone Bipolar Cells and OFF Cone Bipolar Cells

**Figure 5:**
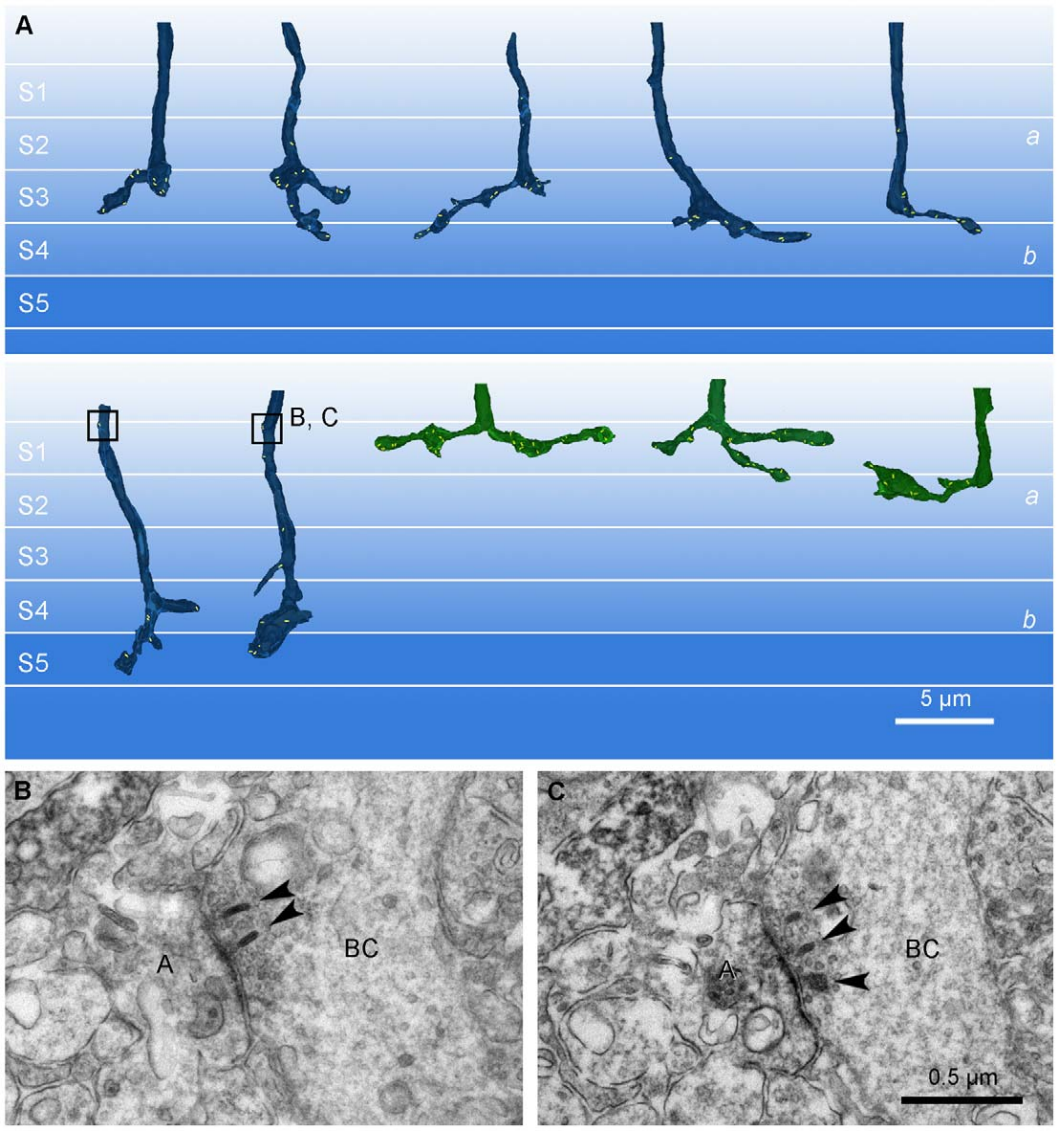
3D reconstruction of calbindin negative ON and OFF cone bipolar cell axons in the IPL. A: Three different types of calbindin negative OFF cone bipolar cells (green) and seven calbindin negative ON cone bipolar cells (slate blue). The ON bipolar cells can be divided into at least 4 different types according to the branching level of the axon terminals. In these cells, only two different ON cone bipolar cells, which ramified in strata 4 and 5, made axonal synapses (boxes) in stratum 1 of the IPL. The second boxed area is shown in B and C at a higher magnification. B, C: These micrographs were taken from two consecutive ultrathin sections. The unlabeled ON cone bipolar axon (BC) makes axonal inputs onto a monadic amacrine process (A). At this axonal synapse, three ribbons (arrowheads) are engaged.

We also reconstructed several non-calbindin bipolar cells (Fig. 5). These included 3 OFF cone bipolar cells, which were stratified in sublamina *a*. They probably comprised different cell types because their terminals stratified at different levels in the IPL. None of the OFF cone bipolar cells made axonal synapses high in sublamina *a* at the INL/IPL border. In fact, none of the OFF bipolar cell axons contained synaptic ribbons at any depth. In contrast, the OFF bipolar axon terminals, below the first branch point, contained numerous synaptic ribbons at post-synaptic dyads, as expected.

Seven, non-calbindin labeled ON bipolar cells were also reconstructed. They all stratified in sublamina *b* and several bipolar cell types were represented because they stratified at different levels. Of these 7 ON bipolar cells, 2 made axonal synapses high in sublamina *a* at the INL/IPL border, which corresponds to the location of calbindin ON cone bipolar axonal synapses, as they passed through sublamina *a* (boxed areas in Fig. 5). Two consecutive serial sections in Fig. 5B show the axonal synapses made monads with multiple synaptic ribbons, very similar to those described above for calbindin bipolar cells. The view of the synaptic ribbons is particularly clear due to the absence of immunolabeled deposits in the bipolar cells. This indicates that several different ON bipolar types make axonal synapses *en passant*.

### Axonal Inputs to ipRGCs

**Figure 6:**
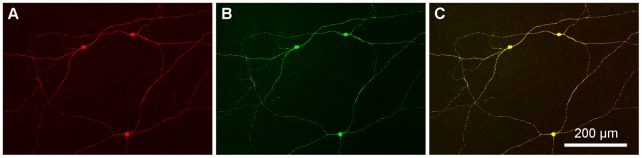
Specificity of melanopsin antibodies. A: Labeling of wholemount rabbit retina with the mouse monoclonal antibody MAB3101 (red). A sparsely branched set of ganglion cells was labeled with the morphological properties of ipRGCs. B: Same frame, wholemount rabbit retina, labeled with the rabbit antibody against the N-terminal of rabbit melanopsin (green). C: Double label image shows exact superposition of the two antibodies (yellow). We conclude both antibodies recognize ipRGCs in the rabbit retina.

**Figure 7:**
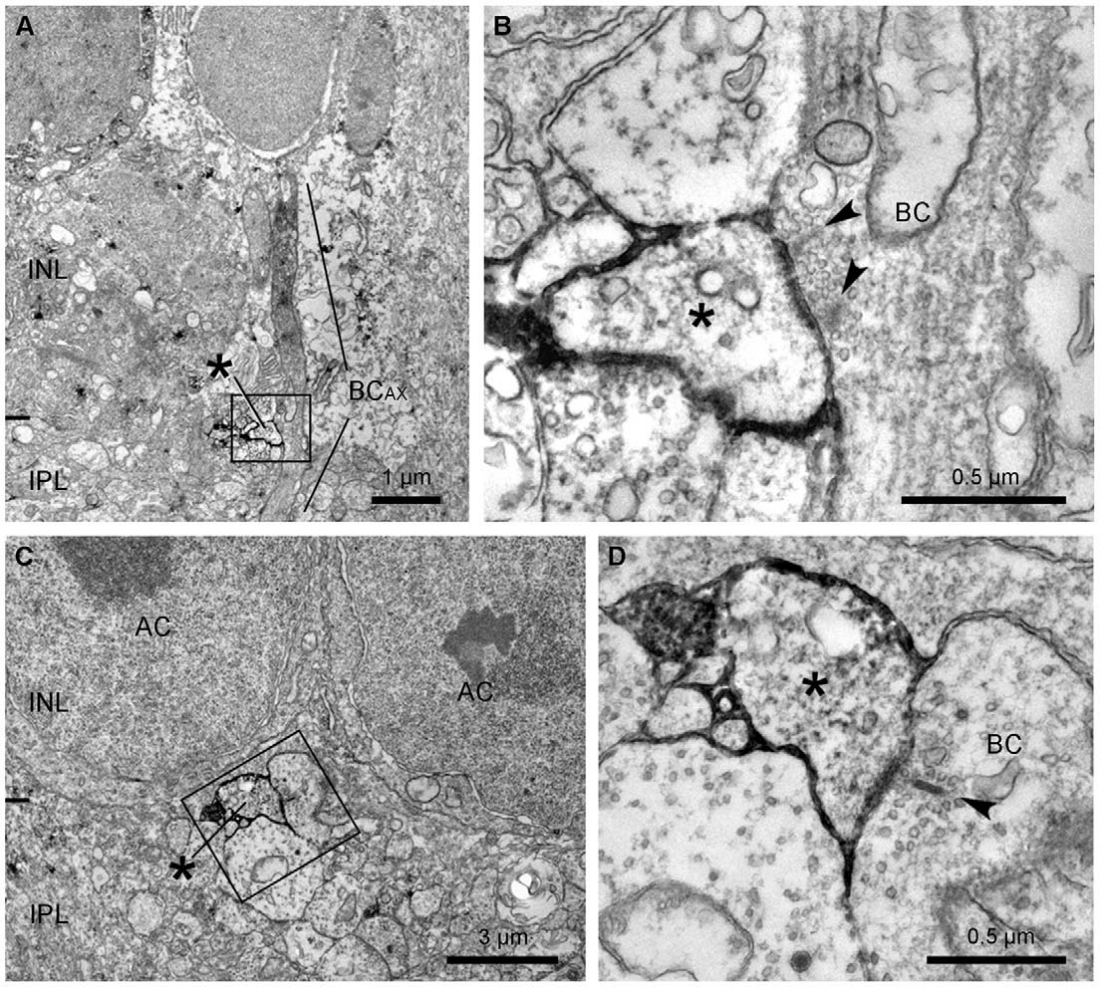
Identification of the synapse between an ipRGC and a putative ON cone bipolar cell in the IPL. A: A low magnification electron micrograph shows the relationship between a labeled ipRGC dendrite (asterisk) and an unlabeled axon of a putative ON bipolar cell (BC_AX_), which is running vertically through the INL and sublamina *a* of the IPL. The boxed area is shown in B at a higher magnification. B: The unlabeled ON bipolar axon (BC) makes an *en passant* ribbon synapse onto a labeled ipRGC dendrite (asterisk). Note that two synaptic ribbons (arrowheads) are engaged in this monadic synapse. C: A low magnification electron micrograph illustrates a labeled ipRGC dendrite (asterisk) located just below the somata of amacrine cells (AC). The boxed area is shown in D at a higher magnification. D: A labeled dendrite (asterisk) makes a postsynaptic monad at the ribbon synapse (arrowhead) from the axon of a presumed ON bipolar cell (BC).

Using immuno-EM combined with a commercial antibody (MAB3101; Millipore) (see Materials and Methods) which fortuitously labels the ipRGCs in the rabbit retina ([26]; Fig. 6, S2), we attempted to find axonal synapses made with ipRGCs in sublamina *a* (Fig. 7). Occasionally, immunolabeled dendrites were located running just underneath the border of the INL. This is the level at which the M1 is stratified. A low magnification image (Fig. 7A) shows a labeled profile, marked with an asterisk. Immediately adjacent, there is an unlabeled bipolar axon, which runs vertically through the INL. The boxed area is shown at higher magnification in Fig. 7B and it reveals that the bipolar axon makes an *en passant* axonal synapse as it passes the ipRGC profile. The ipRGC was the only postsynaptic profile and there were two synaptic ribbons (arrowheads). A second example is shown in Fig. 7C and D. At high magnification, a monad was observed providing input from a single, well-marked synaptic ribbon. This is a small sample, but nevertheless, we have demonstrated that the axonal input to ipRGCs has the same characteristics described for other axonal ribbon synapses.

## Discussion

We used immunoelectron microscopy combined with 3D reconstruction to investigate the ultrastructure of bipolar cells in the rabbit retina. We found numerous examples of the recently reported ectopic or axonal synapses made by ON cone bipolar cells, including but not restricted to calbindin labeled bipolar cells, as they descend through sublamina *a* of the IPL. This layer, immediately adjacent to the INL and coincident with the lamination of the dopaminergic plexus and ipRGCs, constitutes an additional accessory ON sublayer in the outer IPL [25], [26]. The bipolar cell synapses in this layer were characterized by a monadic structure, multiple ribbons and a large postsynaptic density. These may be the diagnostic features of ON bipolar input to DACs and ipRGCs in sublamina *a*.

### Characteristics of the Axonal Synapses

**Figure 8:**
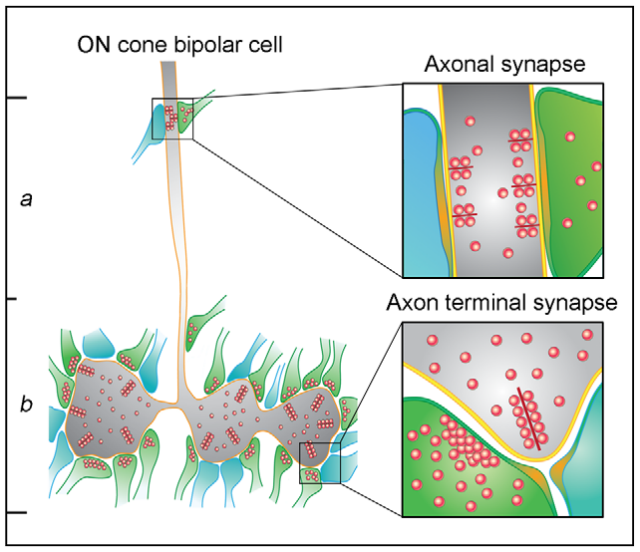
Cartoon summary. Axonal synapse vs. axon terminal synapse of the ON cone bipolar cell. Diagram shows the differences in character between the axonal synapse and the axon terminal synapse of the ON cone bipolar cell in the mammalian retina. Post-synaptic elements are represented as green, for amacrine cells, and blue, for ganglion cell dendrites. The summary shows that axonal ribbon synapses are: 1) monadic, with a single post-synaptic process; 2) they have multiple synaptic ribbons; 3) they have shorter synaptic ribbons and 4) a much larger area of post-synaptic density, when compared to the dyads made by axon terminal synaptic ribbons.

In this report, we found that all of the reconstructed calbindin bipolar cells (10/10) made axonal synapses. This is comparable to a previous confocal report where 88% of a much larger sample of calbindin bipolar axons contained a synaptic ribbon [26]. We found several characteristics of axonal ribbon synapses in sublamina *a* which distinguished them from the axon terminal ribbon synapses in sublamina *b*. The main features of both types are illustrated in Fig. 8.

First, and perhaps most importantly, the axonal synapses were monadic. The descending axons of 10/10 calbindin bipolar cells formed axonal ribbon synapses in the outer IPL that were exclusively monadic (Fig. 2C, S1; Table 1). Other, calbindin-negative, ON bipolar cells did not always make axonal synapses but when they did they were also monadic (Fig. 5). The postsynaptic targets included both dopaminergic and ipRGC dendrites [25], [26] and the input to these cell types in stratum 1 occurred only at postsynaptic monads (Fig. 7). This anatomical arrangement is quite different to the well-described bipolar cell ribbons where the output is directed to a pair of postsynaptic targets, which may be amacrine cells and/or ganglion cells (Fig. 3D). This arrangement is referred to as a dyad. Taken together, both DACs and ipRGCs appear to receive input signals from ON cone bipolar cells *via* synaptic monads, and the monadic ribbon synapse may be a diagnostic feature of ON cone bipolar output in sublamina *a*. It is satisfying that the ultrastructure first described for bipolar inputs to DACs has now been associated with the axonal ribbons shown to provide ON input to dendrites in the OFF layer.

Bipolar cell input to DACs of primate and rabbit retina was first established by Hokoç and Mariani [23], [24]. However, the source of the bipolar input was unknown. They also reported that bipolar cell input in sublamina *a* occurred at dyads or monads but they did not report the presence of multiple synaptic ribbons. It may be that the geometry of a monad is much easier to appreciate when multiple ribbons are detected, as in this work. In the mouse retina, the bipolar inputs to DACs were described as monads [34]. DACs in the mouse retina also have descending dendrites that stratify in stratum 3. Because they are located in sublamina *b*, these may be the recipients of ON bipolar cell input in the usual way [35]. This does not represent a conflict: it is possible that both sites contribute to the ON responses of DACs [35].

Secondly, the axonal or ectopic synapses of ON cone bipolar cells contained multiple synaptic ribbons. In this study, about 90% (89%) of axonal synapses made by calbindin bipolar cells in sublamina *a* involved two or more synaptic ribbons. On average, ≈2.4 synaptic ribbons were present at axonal synapses. A similar finding was previously reported by Calkins et al. [36] who found the descending axon of a blue cone bipolar cell made multiple ribbons onto an unknown ganglion cell monad in sublamina *a* of the IPL in primate retina. From the location, at the border with the INL, plus the presence of multiple synaptic ribbons, this structure appears to be an axonal ribbon synapse, although this was not appreciated at the time. In previous confocal work, most axonal synapses were noticeably larger than other ribbon synapses and they were referred to as giant puncta [26]. This is probably due to the slightly blurred and fused appearance of multiple ribbons in the confocal microscope. Approximately 86% of the calbindin bipolar axons contained these giant puncta and this is close to the number containing 2 or more synaptic ribbons. Multiple synaptic ribbons at axonal synapses made by several ON cone bipolar cell types in sublamina *a* were also reported in a reconstruction of the rabbit retina by Lauritzen et al. [37].

Thirdly, compared to the synaptic ribbons found at bipolar cell axon terminals in sublamina *b*, the axonal synaptic ribbons were short. In the present study, the maximal length of individual axonal synaptic ribbons was 54% and the surface area was 20% of the value for synaptic ribbons at bipolar cell axon terminals. It is possible that the small size of the synaptic ribbons is related to the narrowness of the descending axons. However, with 3.4 ribbons per axonal synapse (reconstructed group, Table 2), the total ribbon surface area was 70% of the axon terminal value. Fourth and finally, at axonal ribbon synapses, the area of the postsynaptic density was more than 5× (582%) that of bipolar terminal ribbon synapses. Compared to bipolar terminal synapses, this big mismatch between presynaptic element and postsynaptic area at axonal synapses could be compensated by the presence of multiple ribbons at each axonal synapse. In this sample of 7 axonal synapses (Table 2), there was an average of 3.4 ribbons. Also at axon terminal synapses, there were two postsynaptic elements. Normalizing to the total area per ribbon yields 86% for axonal ribbons vs. axon terminal ribbons. These numbers are much more comparable. It also suggests there is a relationship between ribbon number and the area of the postsynaptic density. Axonal synapses make monads with much greater postsynaptic areas per synapse but they also have multiple ribbons.

### Several ON bipolar Cell Types make Axonal Synapses

We have confirmed previous confocal work showing that calbindin bipolar cells in the rabbit retina are a major source of axonal synapses in sublamina *a*. However, the reconstructions of other unstained bipolar cells indicate that several bipolar cell types make axonal synapses. None of the OFF bipolar cells described here made axonal synapses. Nor did OFF ganglion cells receive axonal inputs [26]. However, 2/7 additional ON bipolar cells made axonal synapses onto unidentified targets, either amacrine cells or ganglion cells (Fig. 5). The two bipolar cells which made axonal synapses stratified deep in sublamina *b*, at a similar depth to the calbindin bipolar cells, straddling the strata 4/5 border. None of the other ON bipolar cells, which stratified in the upper levels of sublamina *b*, made axonal synapses.

In the rabbit retina, fully half of the giant puncta associated with DACs, were not colocalized with calbindin bipolar cells. Likewise, approximately 75% of the axonal inputs to the postsynaptic spines of bistratified diving cells contained synaptic ribbons, yet were not associated with calbindin bipolar cells [26]. In the mouse retina, ON bipolar cells making axonal synapses resembled type 6 from the catalog of Wässle et al. [38] and type 8 bipolar cells have some swellings and extensions in sublamina *a*, making them likely candidates [8], [25]. In the primate retina, blue cone bipolar cells made axonal synapses in sublamina *a* onto an unknown target [36]. The stratification of the small blue/yellow bistratified ganglion cells was clearly at a different level so these ectopic synapses likely provide axonal input to other cell types at this level, namely ipRGCs and DACs as described in the present work. Presuming these connections hold across mammalian species, this suggests that blue cone bipolar cells may be an additional source of axonal synapses. Thus, the accumulated evidence indicates that several ON bipolar cell types make axonal synapses as they pass through sublamina *a*. Whether different bipolar cells contact different targets in sublamina *a* will require further investigation.

### Functional Considerations

The unique structure of axonal synapses suggests a specialized function. The small ribbon size may be related to the location. The descending axon of the bipolar cell is like a thin pipe with narrow space trafficking many neuroactive substances and cell organelles from the soma to the axon terminal and in reverse. Perhaps this produces a requirement for short ribbons that will not impede microtubular transport. In contrast, axon terminal ribbons in sublamina *b* are located in a relatively spacious terminals packed with synaptic vesicles.

However, the presence of multiple ribbons means that axonal synapses have a multiple release sites and a bigger readily releasable pool of synaptic vesicles synapse [39], [40]. Assuming that a broad postsynaptic density means more transmitter receptors [41], our results imply that axonal ribbons may be very effective synapses. This may be useful because the number of axonal synapses in sublamina *a* is small compared to the axon terminal outputs in sublamina *b*.

### Axonal Ribbon Synapses Provide Input to ipRGCs

In the past 10 years, one of the most studied cell types in the mammalian retina is the ipRGC, a novel type of ganglion cell, which expresses the photopigment melanopsin, and is therefore intrinsically sensitive to light [16], [42]. They form neural pathways to control circadian photoentrainment and the pupillary light reflex [16], [43], [44]. In addition to their intrinsic photorespnses, ipRGCs also receive input from rods and cones *via* bipolar cells [18], [45]–[49]. The ipRGCs are thought to comprise several subtypes according to their morphology [48], [50]–[53]. Among them, M1 cells have ON responses to light, regardless of their stratification in sublamina *a* of the IPL [18], [50], [51], [54]. Thus, along with DACs, the ipRGCs break stratification rules of the IPL. This puzzle was solved by the recent demonstration that axonal synapses form certain ON bipolar cells provide input to ipRGCs and DACs as they pass through sublamina *a* [25], [26].

We were able to locate a total of 4 axonal inputs to ipRGCs from unstained profiles presumed to be ON cone bipolar cells (Fig. 7). At high magnification, they had similar properties to the axonal synapses described above, including multiple synaptic ribbons and a single postsynaptic density. Many bipolar cells must run through sublamina *a* without coming close to an ipRGC dendrite due to their sparse branching pattern. Previous confocal analysis showed that only 6% of calbindin bipolar cells made axonal synapses with ipRGCs compared to 75% for the much denser dopaminergic plexus [26]. In addition, ipRGCs account for less than 1% of ganglion cells in the rabbit retina. These sampling difficulties precluded further analysis but, nevertheless, we have confirmed that ipRGCs receive axonal input in sublamina *a* from axonal ribbon synapses with a common architecture.

## Materials and Methods

### Ethics Statements

This study was carried out in strict accordance with the recommendations in the Guide for the Care and Use of Laboratory Animals of the National Institutes of Health (NIH Publications No. 80–23) revised 1996. The protocol was approved by the IACUC (Institutional Animal Care and Use Committee) in College of Medicine, The Catholic University of Korea (Approval Number: CUMS-2011-0090-01). All surgery was performed under ketamine and xylazine anesthesia, and all efforts were made to minimize suffering.

### Tissue Preparation

New Zealand white rabbits weighing 2.0–2.5 kg were deeply anesthetized with ketamine (100 mg/kg) and xylazine (20 mg/kg). The eyes were enucleated, and the animals were euthanized with an overdose of anesthetic.

The anterior segments of the eyeballs were removed, and the retinas were carefully dissected. The eyecups were fixed by immersion in 4% paraformaldehyde in 0.1 M phosphate buffer (PB: pH 7.4), for 2–3 h. Following fixation, the retinas were carefully dissected and transferred to 30% sucrose in PB for 24 h at 4°C. They were then frozen in liquid nitrogen, thawed, and rinsed in 0.01 M phosphate buffered saline (PBS: pH 7.4).

### Immunohistochemistry

Fifty micrometer-thick vertical and oblique vibratome sections and wholemounts were used. The retinas were incubated in 10% normal donkey serum (NDS) and 1% Triton X-100 in PBS for 2 h at room temperature, to block any nonspecific binding sites. They were then incubated with a monoclonal antibody to calbindin D-28K (Swant, Bellinzona, Switzerland; dilution 1∶3000) or a commercial monoclonal antibody to connexin 45 (MAB3101; Millipore, Temecula, CA; dilution 1∶2000), which fortuitously labels the ipRGCs in the rabbit retina (Hoshi et al., 2009; see below), in PBS containing 0.5% Triton X 100 for 1 day at 4°C. The sections were washed in PBS for 45 min (3×15 min), incubated in the presence of Cy3-conjugated donkey anti-rabbit IgG (Jackson Immuno Research, West Grove, PA; dilution 1∶100) for 2 h, and then washed three times in PBS for 45 min (3×15 min). After thoroughly washing with PB, the retinal tissues were mounted with Vectashield H-1000 (Vector Laboratories, Burlingame, CA).

For double-labeling, sections and wholemounts were incubated for 1 day or 5 days in the mixture of a rabbit polyclonal anti-calbindin antibody and a monoclonal RIBEYE (CtBP2) antibody (BD Transduction Laboratories, San Jose, CA; dilution 1∶2000), respectively. The sections and wholemounts were rinsed for 30 minutes with PBS, and incubated in the mixture of two secondary antisera, a donkey anti-rabbit conjugated with Cy3 (Jackson Immuno Research) and a donkey anti-mouse conjugated with Alexa Fluor 488 (Molecular Probes, Eugene, OR) at a dilution of 1∶200 in PBS containing 0.5% Triton X-100 in PB at 4°C for 2–4 h. After rinsing several times in PBS, the fluorescent specimens were mounted with Vectashield mounting media.

Digital images (1,024×1,024 pixels) were acquired by using a Zeiss LSM 510 Meta confocal microscope (Carl Zeiss Co., Ltd., Oberkochen, Germany) and exported into Adobe Photoshop (Adobe Systems, San Jose, CA). Adjustments were made to stretch the histogram of pixel intensities to fill the entire 8 bit range.

### Melanopsin Antibodies

A commercial mouse antibody, MAB3101 (Millipore), nominally against Cx45, stains a set of ganglion cells in the rabbit retina with sparse dendritic trees. Based on their unique morphology, these ganglion cells were identified as melanopsin ganglion cells or ipRGCs [26]. No other ganglion cell types have such a sparse branching pattern. The reason for the labeling of ipRGCs by MAB3101 is unknown. MAB3101 does not stain Cx45 gap junctions in the rabbit retina, although it has been used to label Cx45 gap junctions in mouse retina.

To confirm the identity of the labeling pattern as ipRGCs, we raised a rabbit polyclonal antibody to a 17 residue peptide from the N-terminal sequence of rabbit melanopsin (MNSPWGSRVPPGPAQEP) (Bethyl Laboratories, Montgomery, TX). The terminal sequences for melanopsin vary across mammalian species so that antibodies against mouse or human sequences do not work in rabbit retina. Labeling the rabbit retina with this melanopsin antibody stained the same sparsely branched ganglion cells. Double labeling with the rabbit melanopsin antibody and MAB3101 produced identical double labeling of ganglion cells with the morphological properties of ipRGCs (Fig. 6). The brightest cells, identified as M1 ipRGCs, were stratified in sublamina *a* (Fig. S2) but several morphological types were present, as reported for mouse retina [51], [52]. We conclude that the rabbit N-terminal melanopsin antibody and the mouse antibody MAB3101 label the same structures, ipRGCs, in the rabbit retina. We used both antibodies interchangeably but it was convenient to use MAB3101 for double label experiments with the rabbit antibody against calbindin.

### Electron Microscopy

Retinal sections were prepared, as described above. After blocking, the sections were incubated in an anti-calbindin or an anti-connexin 45 solution at 4°C for 1 day as used for light microscopy but without Triton X-100. The following immunocytochemical procedures were carried out at room temperature. The sections were washed in PBS for 45 min (3×15 min), incubated in biotin-labeled goat anti-mouse IgG (Jackson Immuno Research; dilution 1∶100) for 2 h, and then washed three times in PBS for 45 min (3×15 min). The sections were then incubated in an avidin-biotin-peroxidase complex (ABC) solution (Vector Laboratories) for 1 h, washed in 0.1 M Tris buffer (TB: pH 7.6), and then preincubated in 0.05% 3,3′-diaminobenzidine tetrahydrochloride (DAB) in TB for 10 min, followed by incubation in the same solution containing 0.05% hydrogen peroxide (H_2_O_2_) for an additional 10 min. The reaction was monitored using a low-power microscope and was stopped by replacing the DAB and H_2_O_2_ solution with TB.

Stained sections were post-fixed in 1% glutaraldehyde in PB for 1 h and, after washing in PB containing 4.5% sucrose for 15 minutes (3×5 min), they were post-fixed in 1% OsO_4_ in PB for 1 h. Afterwards the sections were rewashed in PB containing 4.5% sucrose and dehydrated in a graded series of alcohol. During the dehydration procedure, they were stained *en bloc* with 1% uranyl acetate in 70% alcohol for 1 h, then transferred to propylene oxide, and flat embedded in Epon 812. After curing at 60°C for 3 d, well-stained areas were cut out and attached to an Epon support for further ultrathin sectioning (Reichert-Jung, Nuβloch, Germany). Serial ultrathin sections (70–90 nm thick) were collected on single slot grids (2×0.4 mm), and examined using JEM 1010 electron microscope (JEOL, Tokyo, Japan).

### 3D Reconstruction

For 3D reconstruction, consecutive sections (on average 36 sections per calbindin labeled axon) including labeled axons in sublamina *a* of the IPL were photographed with a digital SC1000 CCD camera (Gatan Inc., Pleasanton, CA). Individual axons were traced with black by using such image processing tools as brush and quick selection on Adobe Photoshop (Adobe Systems) to make a clear distinction from adjacent structures. Then, we aligned each section image along an arbitrary axis by using image movement and rotation to facilitate reconstruction. The 3D models of axons were made transparent to display the synaptic ribbons.

Structures such as pre- and post-synaptic processes, synaptic ribbons, and postsynaptic densities, were masked to make a 3D model of each electron-microscopic image. The masked image was separated into the images containing each structure, and then exported to a 3D modeling program Mimics (version 10.0; Materialise, Leuven, Belgium) along with information on pixel size, slice thickness, and orientation. The boundary of the structures in each image was selected by a thresholding method and then the 3D model was calculated and reconstructed. The positions of processes and synaptic structures in the 3D model were confirmed by projecting the 3D model onto the stack of original electron microscopic images in transparency and clipping mode.

### Measurement of Surface Areas

To measure the area of the synaptic ribbon plate and the postsynaptic density, the reconstructed 3D model was transformed into a stereolithography (STL) file format, which is widely used for rapid prototyping and computer-aided manufacturing. It was exported to a 3D image analysis program, Rapidform XOR (version 3.0; INUS Technology, Seoul, Korea), and then the area of the synaptic ribbon plate and the postsynaptic density were measured.

## Supporting Information

Figure S1 **Examples of the axonal synapse of the calbindin ON cone bipolar cell.** Electron micrographs showing the synapses formed at calbindin bipolar descending axons in sublamina *a* of the IPL. A, B: These photographs were taken from two consecutive ultrathin sections. The labeled bipolar axon gives synaptic inputs onto two ganglion dendrites (G1 and G2) and an amacrine process (A). At these ribbon synapses, all postsynaptic targets are monads and three ribbons (arrowheads) contribute to each synapse. Small white arrowheads in A indicate a ribbon in transport. C: A labeled calbindin bipolar axon descended by an amacrine soma (AC) to form a ribbon synapse (arrowheads) onto a ganglion dendrite (G). AC_N_ and AC_C_ indicate amacrine cell nucleus and cytoplasm, respectively. D: A labeled descending bipolar axon makes an axonal ribbon synapse (arrowheads) onto an amacrine process (A). The amacrine process gives a synaptic input back onto the labeled bipolar axon in reciprocal manner (curved arrow). In this figure, a ribbon in transport (small white arrowheads) is seen in the middle of the axon parallel to the axonal membranes.
(TIF)

Figure S2 **ipRGCs labeled with a commercial antibody MAB3101 in the rabbit retina.** A: Photo-montage of a confocal image taken from an area near the visual streak of a wholemount preparation of the rabbit retina processed for melanopsin immunoreactivity. The image was stacked from the proximal part of the INL through the GCL. Each ipRGC soma is located far from its neighbors, and the long and sparsely branched dendrites appear to be connected in a sparse meshwork. B, C: X-z projection images of confocal stack images taken from retinal wholemounts. Anti-choline acetyltransferase (ChAT) antibody (blue) was used to mark OFF and ON bands formed by conventional and displaced starburst amacrine cells, respectively. B shows a conventional ipRGC type and C shows a displaced type of ipRGC. Both are stratified in stratum 1 of the IPL, above the OFF cholinergic band. Arrows point to ipRGC axons.
(TIF)
