# Supplementary figures and images for: Axonal Synapses Utilize Multiple Synaptic Ribbons in the Mammalian Retina
Source: PLoS One. 2012 Dec 17;7(12):e52295. doi: 10.1371/journal.pone.0052295 (PMC3524110; doi:10.1371/journal.pone.0052295)

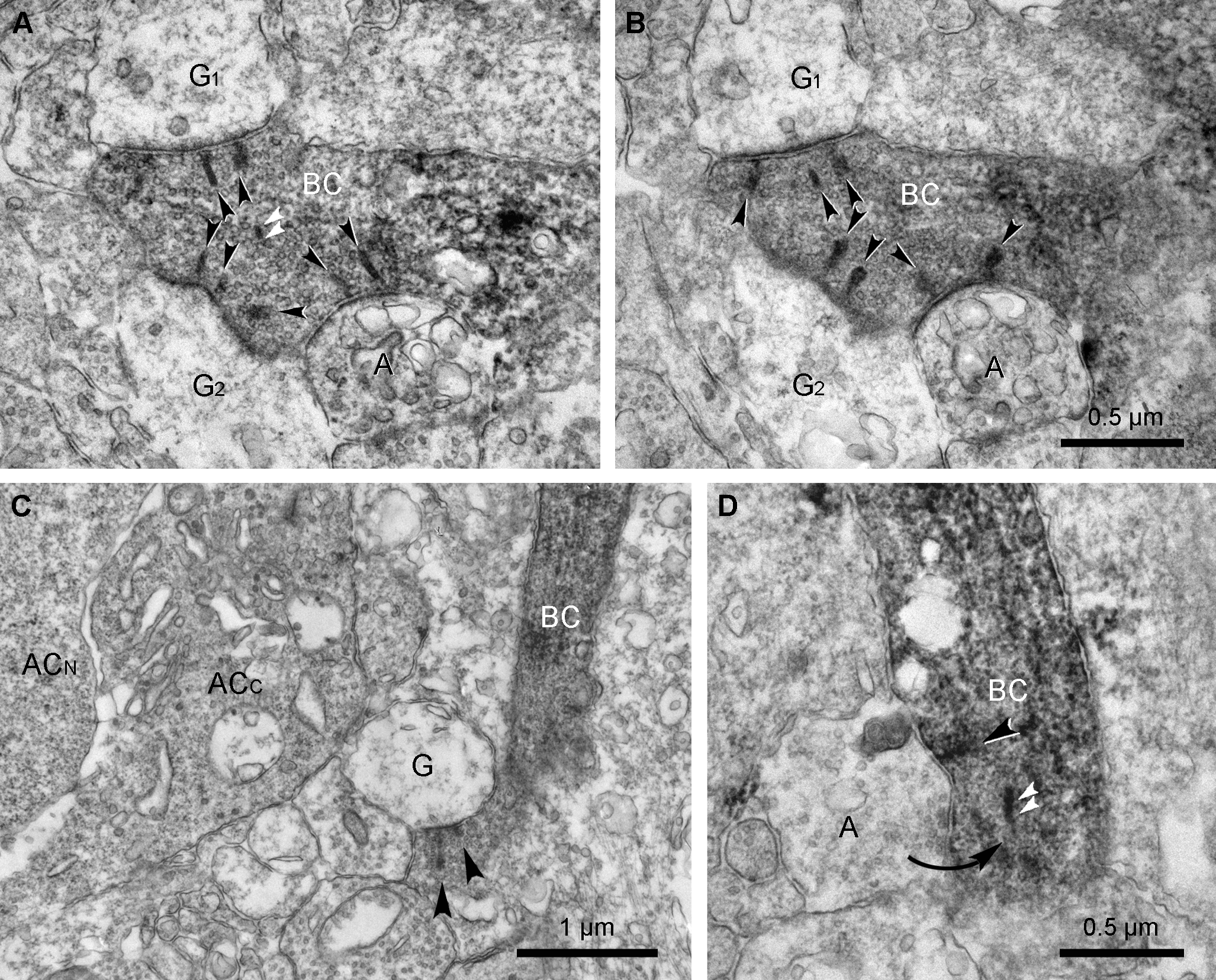

Supplement: Figure S1 — Examples of the axonal synapse of the calbindin ON cone bipolar cell. Electron micrographs showing the synapses formed at calbindin bipolar descending axons in sublamina a of the IPL. A, B: These photographs were taken from two consecutive ultrathin sections. The labeled bipolar axon gives synaptic inputs onto two ganglion dendrites (G1 and G2) and an amacrine process (A). At these ribbon synapses, all postsynaptic targets are monads and three ribbons (arrowheads) contribute to each synapse. Small white arrowheads in A indicate a ribbon in transport. C: A labeled calbindin bipolar axon descended by an amacrine soma (AC) to form a ribbon synapse (arrowheads) onto a ganglion dendrite (G). ACN and ACC indicate amacrine cell nucleus and cytoplasm, respectively. D: A labeled descending bipolar axon makes an axonal ribbon synapse (arrowheads) onto an amacrine process (A). The amacrine process gives a synaptic input back onto the labeled bipolar axon in reciprocal manner (curved arrow). In this figure, a ribbon in transport (small white arrowheads) is seen in the middle of the axon parallel to the axonal membranes. (TIF) [file pone.0052295.s001.tif]

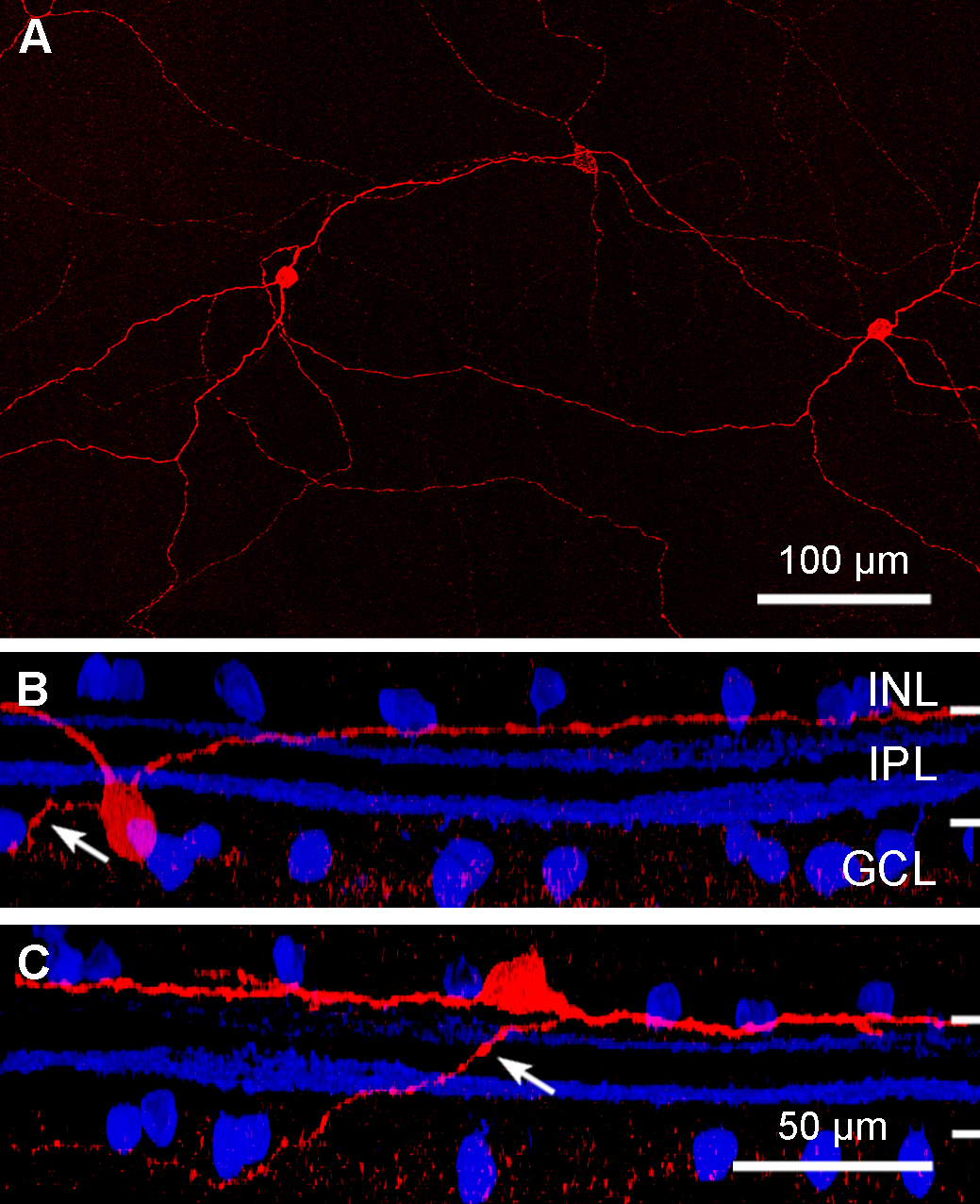

Supplement: Figure S2 — ipRGCs labeled with a commercial antibody MAB3101 in the rabbit retina. A: Photo-montage of a confocal image taken from an area near the visual streak of a wholemount preparation of the rabbit retina processed for melanopsin immunoreactivity. The image was stacked from the proximal part of the INL through the GCL. Each ipRGC soma is located far from its neighbors, and the long and sparsely branched dendrites appear to be connected in a sparse meshwork. B, C: X-z projection images of confocal stack images taken from retinal wholemounts. Anti-choline acetyltransferase (ChAT) antibody (blue) was used to mark OFF and ON bands formed by conventional and displaced starburst amacrine cells, respectively. B shows a conventional ipRGC type and C shows a displaced type of ipRGC. Both are stratified in stratum 1 of the IPL, above the OFF cholinergic band. Arrows point to ipRGC axons. (TIF) [file pone.0052295.s002.tif]
